# South African adolescents' lived experiences of acquired hearing loss following multidrug-resistant tuberculosis treatment

**DOI:** 10.3389/fresc.2024.1336346

**Published:** 2024-02-26

**Authors:** Tarryn Sparg, Lucretia Petersen, Pat Mayers, Christine Rogers

**Affiliations:** ^1^ Division of Communication Sciences and Disorders, Faculty of Health Sciences, University of Cape Town, Cape Town, South Africa; ^2^ Division of Nursing and Midwifery, Faculty of Health Sciences, University of Cape Town, Cape Town, South Africa

**Keywords:** hearing loss, MDR-TB, ototoxicity, phenomenology, young adults, patient-centred care

## Abstract

**Objective:**

The impact of acquiring hearing loss might be exacerbated during adolescence, as this normal transition from childhood to adulthood is characterised by identity construction and social intensity. This study aimed to describe the lived experiences of South African adolescents with acquired hearing loss following aminoglycoside treatment for multidrug resistant tuberculosis.

**Design:**

The study adopted a descriptive phenomenological design and in-depth, semi-structured interviews were conducted in English, isiZulu and Afrikaans. The data was managed and analysed according to a modified version of Hycner's framework.

**Study sample:**

Six participants aged 16–24 years with bilateral, mild to profound hearing loss acquired from aminoglycoside treatment were recruited from two South African provinces.

**Results:**

Three themes emerged which created a triple burden for participants. They endured socio-economic hardship encompassing limited economic and emotional support. Participants battled the consequences of life-threatening MDR-TB including illness, hospitalisation, stigma, and other challenges. Finally, participants were left with life-changing hearing loss.

**Conclusion:**

The findings indicate the necessity of holistic management of adolescents with aminoglycoside-related acquired hearing loss and serves as motivation to improve ototoxic monitoring practices and patient uptake of monitoring services and calls for the cessation, or at least cautious use, of aminoglycosides.

## Introduction

Each year, approximately 850,000 adolescents (between 10 and 19 years) and around 1 million young adults (20–24 years) are diagnosed with TB (1); with one in five succumbing (2). Worldwide, most adolescents and young adults (AYA) live in low-and middle-income countries (LMIC), thus young people form a considerable percentage of these regions' general population, and in high-TB burden countries including South Africa, a notable proportion of patients with TB (3). The HIV epidemic has been the main contributor to the rise of TB in South Africa (4), as HIV infection raises the risk of developing active TB between 20 and 30 times (5). The susceptibility to TB in HIV positive individuals is due to reactivation of latent TB; the greater risk of infection once exposed to TB (re-infection), and more rapid progression of TB soon after infection (5). In AYA with TB and HIV, the HIV status of the patient, whether on anti-retrovirals or not, substantially raises the likelihood of death within six months of the TB diagnosis (6).

LMIC with challenges in health care systems and delivery, along with poverty, present settings which promote the transmission of TB (5). Multi-drug resistant TB (MDR-TB), which emerged as the result of poor patient adherence and poor management of patients and drug supply, has complicated the management of TB (7). However, the role of HIV infection should not be overlooked as a risk factor for developing MDR-TB, as such individuals have a much higher risk for the development of a resistant form of TB (8).

The current MDR-TB treatment regimen has recently been updated, but still includes medication with permanent side-effects administered under directly observed therapy (DOT) (9). Aminoglycosides, injectable agents such as amikacin and streptomycin used in the regimen, are specifically associated with ototoxicity (10). Ototoxicity refers to the irreversible damage to the cochlea and vestibular organs and is associated with permanent hearing loss, tinnitus and dizziness (11). Reports regarding the occurrence of hearing loss among MDR-TB patients receiving aminoglycosides vary due to differing drugs and drug regimens, methodology and definitions and quantification of hearing loss, but recent meta-analyses cited 28.3% (95%CI: 23.4–33.3) (12) and 40.62% (95%CI: 32.77–66.61) (13). Of note, a South African study suggested that risk factors for exacerbating the risk of ototoxicity included HIV status, malnutrition, initial dosing, and pre-existing hearing loss (14); suggesting the risk is more acute in LMIC. Despite the variation in statistics, aminoglycoside-related acquired hearing loss in South Africa warrants concern considering the risk due to the MDR-TB burden.

The negative psychological, social and employment consequences associated with acquired hearing loss in adults have been well established (15), albeit not in the context of life-threatening MDR-TB which is associated with its own significant reduction in social, occupational and quality of life dimensions (16–18). However, it is reasonable to argue that the sequalae of ototoxic hearing loss are worse in LMIC, due to limited hearing health care and rehabilitation (13).

Ototoxicity within the context of adolescence has not been explored in depth (13) and presents an opportunity to study the consequences of acquired hearing loss during this life stage. The impact of acquiring a hearing loss might be exacerbated during adolescence as this normal transition from childhood to adulthood is characterised by rapid development in which values, beliefs and aspirations are constructed and revised in accordance with identity (19). In addition, AYA are particularly vulnerable to the effects of social isolation, hospitalisation and stigma, all of which can compromise their development and have long-term impact (1, 20). Development of increased autonomy, new ideas, relationships with peers and others, can make growing into adulthood a sensitive and challenging period, but also one of rich developmental opportunities (19).

Awareness of the experiences of AYA is crucial as they have unique needs and challenges in general and specific to management of TB, yet they have been largely excluded from approaches to TB (2, 21, 22). Recent calls have been made to focus on the needs of children and adolescents with TB (22). According to the Population Reference Bureau (23), 28% of South Africa's population is below the age of 15 years. Research pertaining to AYA is thus critical in South Africa with such a significant proportion of the population entering adolescence. Knowledge of how this population is affected by acquired hearing loss is important to health practitioners as it has implications for how young patients are managed.

The experiences of AYA can only be studied by taking a qualitative approach. Descriptive phenomenology, a qualitative paradigm, has been used in the study of human experiences as it aims to intricately understand and describe individuals' lived experiences (24). In other words, it aims to investigate a phenomenon as perceived by the individual experiencing that phenomenon. Descriptive phenomenology distinguishes itself from other qualitative and phenomenological designs by its key concepts, adoption of an objective attitude achieved through “bracketing” and its intrinsic methodology.

This study aimed to explore the lived experience of South African adolescents living with acquired hearing loss following aminoglycoside treatment.

## Method

A qualitative, descriptive phenomenological design was adopted. Purposive sampling was utilised to recruit individuals between the ages of 12–24 years with mild to profound, bilateral, sensorineural or mixed hearing loss, acquired after age 12 years following aminoglycoside treatment via hospitals in the Gauteng and Western Cape provinces of South Africa. Six participants were recruited. Data saturation was used determine the number of participants; a sample size of 10 or fewer is typically sufficient to extract the essence of the experience (24).

Ethics approval (HREC #531/2012) was obtained from the University of Cape Town's Faculty of Health Sciences Human Research Ethics Committee. Appropriate informed assent and consent procedures were followed. Information and assent/consent forms were provided in the participant's preferred language.

In-depth, semi-structured interviews, lasting 60–90 min, were conducted. Four participants were interviewed in English by one researcher. One participant was interviewed in isiZulu, and one was interviewed in Afrikaans by trained research assistants fluent in the respective language. Two participants had interview questions asked in writing as they could not hear speech due to the severity of their hearing loss.

**Figure 1:**
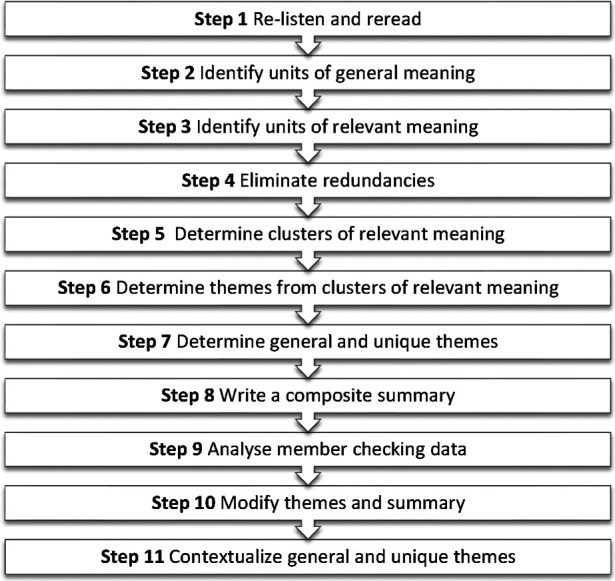
Steps used in data analysis based on Hycner's phenomenological method for analysis (25).

Interviews were audio-recorded and transcribed. Audio-recordings of interviews conducted in isiZulu and Afrikaans were translated into written English by translators and the correctness checked by the research assistants who conducted the interviews. The data were analysed using a modified version of Hycner's framework (25), outlined in Figure 1.

Trustworthiness was ensured through bracketing, a multifaceted process in which the researcher sets aside her preconceptions of the topic, and member checking, in which participants are asked to check the researcher's analysis of their interviews. Additionally, strategies of peer debriefing, an inquiry audit, and thick description were employed to ensure trustworthiness.

The study conformed to the ethical principles in accordance with the Declaration of Helsinki (26). Participants' identities were protected by self-selected pseudonyms and the recruiting sites were not disclosed. The option to be referred to a social worker/psychologist was provided.

## Findings and discussion

Descriptions of participants are detailed in Table 1. Participants experienced a triple burden because of aminoglycoside-related acquired hearing loss; they lived with socio-economic hardship, life-threatening MDR-TB, and life-changing hearing loss, which emerged as three major themes. The themes, subthemes that constituted each theme, and selected quotations, are presented in Table 2.

**Table 1:** Participant descriptions.

Participant (Sex, age)	Degree of hearing loss	Age in years at commencement of aminoglyco-sides	Age in years at onset of hearing loss	Period of hospitalisation	Other medical conditions	Current or completed school grade	Involvement in organised or social activities	Hearing aid	Language preference/communica-tion mode
Elizabeth F, 24	Profound HL bilaterally	18	19	Intermittently since age 18, hospitalised at time of interview	None	Completed Gr. 11	Church	Yes	English/written
Bianca F, 19	Severe HL bilaterally	17	17	None	HIV+	Completed Gr. 11	Church, tennis	Yes	Afrikaans/verbal
Fortunate F, 16	Moderately-severe HFHL bilaterally	13	14	2 mo.	HIV+	Currently Gr. 10	Hockey, peer counsellor	Yes	English/verbal
Sandisile F, 24	Mild HL bilaterally	22	24	7 mo.	HIV+	Completed Gr. 11	Limited	No	IsiZulu/verbal
Liezel F, 22	Moderate HL in right ear, mild HL in left ear	19	20 (2 mo. later)	None	None	Completed Gr. 12	Retail sales assistant, church	Yes	English/verbal
Teekay M, 23	Profound HL bilaterally	22	22 (2 mo. later)	6 mo. previously, hospitalised for 2 mo. at time of interview.	None	Completed Gr. 12	Limited	Yes	English/written

F, female, M, male, HL, hearing loss based on audiometric pure-tone thresholds averaged at 500, 1,000 and 2,000 Hz, HFHL, high frequency hearing loss based on audiometric pure-tone thresholds averaged at 4,000, 6,000, 8,000 Hz, mo., month/s, Gr., school grade.

**Table 2:** Themes and subthemes constituting the triple burden of living with acquired hearing loss following aminoglycoside treatment.

Themes	Subthemes	Selected quotations
Living with socio-economic hardship	Living with limited economic support	*My sister’s husband says, “you have grown up, you have to do your own things”.* Teekay
	Living with limited emotional support	*I am only realizing it now that my maternal family does not love me… Even when I was in hospital they never even came to see me.* Sandisile
Living with life-threatening MDR-TB	Living with illness	*I was thin thin, thin, thin, thin!… I was wearing a [disposable diaper] before and then they put a drip on. I was really sick. And I was unable to walk.* Elizabeth
	Living hospitalised	*^…^ they move you for Friday, Saturday, and Sunday and Monday you go back and on days like Christmas you are allowed to come back.* Sandisile
	Living with treatment challenges and limited awareness of side-effects	*…and later stopped fetching the treatment, because I had to go to the clinic every day.* Bianca
	Living with stigma of TB	*They said, “You should have your own cup, your own spoon, and everything else that you need to use should be your own and you should not use other people’s things” …*Sandisile
	Living with school and employment challenges	*The life I'm living now, you can't get a job nicely… So if you go to work, you work with lots of people, you are not putting a mask on, it’s going to spread.* Teekay
Living with life-changing hearing loss	Living with loss of normal hearing	*And so I told my sister to put the TV louder because I can’t hear what the people are saying. And then she said, “it is already loud, why do you want to put it louder?”* Liezel
	Living with loss of effortless communication	*If someone talks to me, I have to pay attention. When you say something, if I don’t understand, I say, “Come again?” I really think fast what she is trying to say.* Teekay
	Living with fear of permanent, complete hearing loss	*I thought my hearing would have got worse, to a point where I would have had to speak sign language.* Fortunate
	Living with stigma of hearing loss	*I always felt embarrassed when I wore [the hearing aid] …People always looked at me when I was wearing that thing.* Bianca
	Living with others’ reaction to hearing loss	*When people are telling their stories, you try to go there. Then they start to talk, you say, “hey guys, what are you saying?” They keep on continuing with the story, they don’t tell you*. Teekay
	Living with the consequences of hearing loss	*Even at home, I like to isolate myself, I like to sit in my room, watch DVD or TV in my room. I don’t sit with them in the room like the kitchen or dining room, sitting there chatting, I don’t… *Elizabeth

The first theme to emerge was living with socio-economic hardship. Poor social and economic circumstances were found to be common to all but one participant. These factors had significant consequences for how participants experienced MDR-TB and hearing loss.

Living with limited economic support contributed to hardship. Four of the five working-aged participants were unemployed. Unemployment was attributed to several factors including incompletion of schooling, which was the reality for three participants because of pre-existing socio-economic difficulties. In the context of TB, there are no studies on the impact of school absence (3), but being unable to complete secondary school has been explored in South Africa. Consequences include increased levels of poverty, and importantly given the hearing loss in the study participants, ill health including mental health. Particularly if coming from a deprived background, unemployment is likely to last longer and income capacity when employed is less than qualified high school leavers (27). Overall, South African youth are at an economic disadvantage considering that the unemployment rate for South Africans younger than 25-years-old was 33% in the fourth quarter of 2022 (28).

Employment struggles meant that most participants were not financially independent and relied on the limited and sometimes reluctant assistance of others, as noted by Elizabeth's experience:

“*My aunt’s husband said he will pay for me [to go to school], he’s got money, so my aunty said ‘no, I’m not her daughter’.*”

Other MDR-TB patients have reported having to rely on financial support from their family or borrow money from family or friends and even after treatment completion, financial vulnerability persists for TB-affected households (29). In contrast, researchers have highlighted the positive outcomes for TB patients who receive patient-centred care and financial support, including programmes in LMIC (22). Participants' financial circumstances meant that they had to move in with relatives, as other TB patients have been forced to do (30). Participants were not welcomed in their families' homes, leaving them feeling displaced. Teekay expressed that the hospital was the only place he felt he could live:

“*There’s nowhere I can go besides this place (hospital)… When I go to Zimbabwe, nobody wants to stay with me.*”

Lack of economic support and displacement can be part of orphanhood. In this study, four of six participants had lost one or both parents. Given the context (South Africa) and timing of data collection (2013), parental loss requires further interrogation. A longitudinal surveillance programme in rural Kwa-Zulu Natal (31) revealed an increasing proportion of children and adolescents <20 years of age over time, peaking in 2010, with 36% being orphaned with the loss of one or both parents. In 2010, 53% of AYA between the ages of 15–19 years had experienced the death of a parent or both, with HIV/AIDS and TB being the predominant causes of parental death (31). It would be interesting to understand not only the impact of the loss of parents, but the relevance of the cause of death to AYA, particularly with TB.

Research with orphaned AYA in another economically depressed South African province is thought-provoking in unravelling this study's participants' experiences. Consequences of orphanhood were reported to include the understandable bereavement, but also psycho-social challenges including early withdrawal from school, which was a major stressor (32). Loss of parental support exacerbated participants' socio-economic hardship, which had to be negotiated along with their grief. Ntuli and colleagues also reported hunger, increased poverty, reduced support from other family members and ill health as associated with orphanhood. Interestingly in the face of hearing impairment, Ntuli et al., reported participants resorted to silence. Losing a parent was emotional and life-changing:

“*…without my dad I thought my life was over (crying)… ‘Cause he was everything (crying)…*” (Fortunate)

Absent parents, due to neglect and abandonment, similarly compounded issues of socio-economic hardship. Several participants reported how their parents were not active in their lives and neglected their parental duties. Sandisile explained:

“*My mom would refuse to buy me school things like shoes and uniforms.*”

Having living parents was not necessarily synonymous with having loving and supportive parents. Although Elizabeth's father was alive, she felt alienated, and stated “*I don’t have parents*”. Henderson (33) argued that being an orphan in South Africa constitutes “destitution, alienation and a lack of belongingness” (p. 307) and thereby suggested that an orphan is also one who has parents, but has experienced profound displacement, as evident by Elizabeth's account:

“*…my mom passed away when I was young, so even my father, maybe he thinks I’m not his daughter. Sometimes, I ask him those questions because even he doesn’t want me…*”

As discussed, certain South African authors have documented that orphanhood has negative social, emotional, educational and economic consequences (32, 34). Such consequences are also associated with MDR-TB and with hearing loss, as the findings demonstrate, and may be compounded by orphanhood.

Turning now to hardships more directly related to the lived experience of MDR-TB, living with limited emotional support continued as a sub-theme of hardship. Participants felt abandoned by their families while they were hospitalised, as family members seldom made efforts to visit them. Some family members would make excuses when being pleaded with to visit:

“*When I called them, they said, ‘uh I'm busy, I'm still at work’. When I call after work, they don’t answer the phone.*” (Teekay)

AYA depend on family members for emotional support during periods of hospitalisation. The long period of isolation associated with early treatment for TB is particularly trying and challenge AYA's nascent autonomy (35). Without caregivers' comfort and attention, negative emotions and increased experience of social isolation result (1). Family members may not have been present in participants' lives because of stigma related to TB. It is not uncommon for individuals to distance themselves from those with MDR-TB when they are perceived to be infectious (36). As well as physical distancing, emotional distancing was perceived when participants were not treated with compassion and empathy. Some participants were even blamed for their circumstances:

“*They will just criticise me, my other sister will say, ‘I'm not the one who got infected with TB, just stop irritating me, I’ve got my own babies’…*” (Elizabeth)

The availability of empathy and emotional support at home is often lacking for TB and MDR-TB patients, which is detrimental as lack thereof impacts adherence (37). In contrast, a study set in Siberia, found that caregiver and family support of AYA pre-and post-hospitalisation can ameliorate the anxiety and apprehension experienced by AYA coming to terms with TB diagnosis and treatment (35).

Participants expressed feelings of being unloved and rejected because of the physical and emotional abandonment and indifference they experienced:

“*I am only realising it now that my maternal family does not love me.*” (Sandisile)

Such emotions may have been exacerbated by participants' orphaned statuses. Orphans in Saraswat and Unisa's (38) study experienced a longing for love and affection; rejection, and isolation from the world outside of the orphanage akin to the institutions in which participants were hospitalised (38).

The **second** major theme was living with life-threatening MDR-TB. Living with MDR-TB, for which treatment resulted in participants acquiring a hearing loss, was intrinsic to participants' experience of hearing loss.

Participants endured living with illness. They had to bear the physical symptoms of MDR-TB, particularly coughing and excessive weight loss, which are commonly associated with TB (39).

Participants expressed uncertainty about how they contracted their illness. In reaction to their diagnosis, they felt confusion and disbelief:

“*I did not believe that I really had it… I was too confused and sad.*” (Bianca)

Participants' uncertainty may be related to several factors. Around the time this study was conducted research suggested less than half of adults receiving TB treatment in Gauteng province were provided with information about TB, its symptoms and how it is contracted (40). Later South African literature suggests knowledge has improved (41, 42) with 90.1% of MDR-TB patients having received information about MDR-TB from a healthcare worker in Maharaj et al.'s (42) study. More recent research has demonstrated that patients are knowledgeable about TB, risk and prevention (43). Interestingly, the same authors noted that young people have difficulty in understanding that TB is a bacterial infection, particularly in regard to treatment and adherence (43). Pertinent to this study's adolescent population, Chinese high-school students with TB demonstrated poor knowledge of TB, including the cause and transmission of the illness (44). The students were similarly confused by their diagnosis, primarily because they perceived themselves as being in good health (44). On initial diagnosis of TB, patients have expressed being unable to believe the diagnosis (43, 45), like Bianca's statement above. Other responses reported in newly diagnosed TB patient include anger, stress and nervousness (46).

All the participants reflected on living hospitalised. Participants expressed negative and positive aspects to life within an institution. Participants' freedom of movement within the hospitals was restricted to minimise the spread of MDR-TB. Their activities within and outside of the hospitals were strictly regulated:

“*They move you for Friday, Saturday, and Sunday and Monday you go back and on days like Christmas you are allowed to come back.*” (Sandisile)

Restricted movement was the least of Elizabeth's worries, who related her disturbing experience of being mistreated by a nurse:

“*The [nursing] sister last week said she wanted to kill me… So she tied me on the cold bed there, she tied my hands. It’s too painful when I touch my hands…*”

TB patients have reported negative nurse attitudes which affected their adherence to treatment (45, 47). In primary health care facilities in South Africa, TB patients sometimes experienced abusive attitudes by staff, even reporting being sworn and shouted at (48, 49). A review of global literature upheld the notion of poor and stigmatising attitudes from health care workers towards TB patients (50); while other reports noted the uneven power dynamic between patients and their providers (51). Specifically related to nurses working in TB care, work pressures, daily frustrations (52), the stressors of working with a life-threatening infectious disease, eagerness to control patients in an attempt to control the disease, the belief that nurses know what is best for patients, and professional insecurities may compromise nurse-patient interactions (53). Elizabeth's recount may indicate that she had adherence difficulties or was experiencing medication-related psychosis; this cannot be corroborated but could have contributed to the nurse resorting to such inexcusable behaviour. Nurses have expressed anger and frustration about TB patients who do not adhere to treatment (53). Nurses in TB control may be perceived as uncaring in having to navigate being firm and compassionate in managing non-adherence to treatment (52). A South African study investigating the experiences of nurses caring for MDR-TB patients found that nurses felt non-adherence by patients impacted on their ability to provide quality care (54). More recent work (55) has suggested that in Africa, nurse-patient interaction remains poor. Kwame and Petrucka's scoping review of patients' experiences of nursing including in public health settings, revealed that patients' concerns are overlooked; their needs ignored, and abuse, disrespect and humiliation was described during clinical encounters.

While Teekay acknowledged that he “*did… see bad things happen in the hospital…*” he described positive aspects of hospitalisation such as having less self-responsibility:

“*Now when you go out of the hospital, you have to look after yourself.*”

Finally, TB and HIV patients are recipients of nursing bias, which can result in disparate care (56). Groves et al., cited a Ghanaian study in which nurses shunned and maltreated TB patients. Poor socio-economic status formed another group of patients who experienced nursing bias (56).

Separate to the issues of being hospitalised, living with treatment challenges and limited awareness of side-effects were reported by participants. The nature of MDR-TB treatment and the side-effects thereof were particularly challenging. Injectables were administered daily and were painful. The painful nature of injectables has contributed to patients defaulting treatment (47). Participants experienced adverse treatment side-effects. For Elizabeth, whose severe effects including temporary blindness, weakness, tremors, loss of appetite and psychotic behaviour, it felt as if she was going to die:

“*They want to kill me, they’re giving me yellow medication, and much!*”

Adverse side-effects are cited by patients as the most challenging factor affecting adherence (37, 47). Patients with HIV and MDR-TB in an Indian study reported that the side-effects of MDR-TB treatment as worse than the illness itself (57).

It was tempting for participants to stop treatment, particularly as they had to attend the clinics daily for DOT. This resulted in one participant's non-adherence. In addition to adverse side-effects, daily DOT is a significant burden for patients (58) and has been associated with treatment default (37, 47). While DOT facilitates drug administration and MDR-TB control, its inherent challenges were evident in the current study.

All but one participant was unaware of the ototoxic side-effects of aminoglycoside treatment before commencing treatment, which is alarming. Elizabeth captured the lack of information prior to treatment:

“*…The sisters (nursing staff) in the ward didn’t explain anything. So, the patients told me that there are people becoming deaf here because of treatment.*” (Elizabeth).

Similarly, TB defaulters in a Namibian study reported receiving limited information about treatment side-effects from nurses, only being informed after the onset of such effects (47). In a South African study only 6% of adults receiving TB treatment were told about the general side-effects of treatment and only 20% were aware of auditory-related side-effects (40). In another South African study, 32% of medical students had witnessed inadequate health information being given to patients (59). Only 16% of doctors in a South African tertiary hospital, albeit not specifically treating MDR-TB, consistently disclosed ototoxic risks to their patients and 28% never disclosed such risks (60). Lack of disclosure was attributed to high caseloads and time constraints (60).

Besides the physical challenges MDR-TB, living with the stigma of TB was distressing for participants. It is widely reported in the literature that patients with TB and MDR-TB must contend with stigma (47). For example, patients with MDR-TB in Baral et al.'s (58) study reported significant enacted stigma resulting in them being isolated by their families and in a breakdown of family relationships (58). Given that in this study, three of the six participants had HIV, it is important to acknowledge that it is difficult to discuss TB stigma in isolation, due to frequent comorbidity between TB and HIV infection. This dual infection has developed into a double or compound stigma (61) which must be addressed. In South Africa, with a high burden of both diseases, research has shown that individuals tend to deflect stigma away from their HIV status, despite the negative associations with TB infection (20). Another concern for the fragility of adolescence, is the tendency for stigma to be internalised by individuals and promote low self-esteem (20), which of course will be compounded by hearing loss. As a result of the contagious nature of MDR-TB, participants experienced stigma and were ostracised. They described how family members feared being in contact with them, even to the extent of not being allowed to use family members' utensils. The stigma and exclusion participants endured created feelings of sadness and loneliness.

Isolation continued outside the family setting. Participants were labelled negatively by their communities because of their MDR-TB and/or HIV status:

“*At home when I was sick, they used to tell people at (her) home that I’m HIV-positive*”*.* (Elizabeth)

Elizabeth's experience was not unusual, with reports of other MDR-TB patients being subjected to gossiping neighbours, and identified by use of specific healthcare facilities for TB (20). Hatred or avoidance by neighbours and friends (58) frequently results. Discrimination is further intensified because of the misguided notion in communities that TB is always associated with HIV (62). Efforts to “cover” or deflect stigma against HIV are made by some individuals given the notion that TB is regarded as treatable and not the person's fault, while HIV is perceived as permanent and the individual with it is somehow culpable (61).

Considering the stigma participants endured, it is not surprising that they chose not to disclose their illness to close friends:

“*After I came back from hospital my friends said, ‘Why did you not tell us that you had this and that disease because we were told never to ever go drinking with you, and to not use things with you, and you have to use your own things separately.’*” (Sandisile)

In a South African study, most children and adolescents were also selective in disclosing their MDR-TB status (36). It is not uncommon for individuals with MDR-TB to conceal their disease due to fear of stigma and being discriminated against (36), fear of rejection (58) and potential detrimental effects on one's social status in the community (37). Given that TB is associated with poverty, and in South Africa, frequently with HIV, stigmatisation appears to be present at multiple strata of living.

Continuing with participants’ experiences outside of the home, living with school and employment challenges were encountered. For example, one participant failed a school grade in the year that she was receiving treatment, and a second discontinued schooling because of her illness. Illness was among the top three reasons why female scholars in a rural province of South Africa dropped out of high school (63). It is common for students with MDR-TB to experience educational challenges; lengthy absenteeism made returning to school particularly challenging for children in Franck et al.'s (36) study (36). High-school students with TB have reported that having to discontinue school was the biggest challenge of their illness (44).

MDR-TB is often a source of employment difficulties (57, 58, 64). Studies from Nigeria and China have linked MDR-TB to job loss or inability to work, with the Chinese study reporting more than half their 161 participants had lost their jobs (65, 66). Adverse treatment effects and the daily TB treatment regimen have affected patients' ability to sustain their livelihood (37). Individuals must take time off work which results in losses in income (37). Often they stop working altogether (57, 58, 64) and have difficulties finding work thereafter (58). Those who continue to work often experience employers' dissatisfaction as their job performance is diminished (58).

MDR-TB was only reported as a specific barrier to employment by Teekay, who was the only unemployed participant who had completed his schooling and therefore attributing his employment challenges to illness. Other participants attributed their difficulties to lack of education. Teekay was concerned about spreading MDR-TB to others in the workplace. He also felt that adverse side-effects and daily trips to the clinic impacted on his ability to obtain a job and would make maintaining a job difficult, reflecting the literature above.

The final theme was *living with life-changing hearing loss.* Participants were left with permanent hearing loss as a reminder of their temporary, albeit potentially life-threatening illness.

Living with loss of normal hearing emerged as the first subtheme. Tinnitus, which affected all but one participant, was an initial indicator of hearing disturbances, which is typically one of the first symptoms of ototoxicity (67). The tinnitus was intrusive, with two participants describing it as painful. In a South African study, most adults receiving TB medication, who reported that auditory symptoms consistently affected their daily lives, experienced hearing loss and/or constant tinnitus (40).

The initial hearing deterioration was a difficult and confusing time for participants who “*[were] fine and hearing but now suddenly, unexpectedly, have hearing problems*”. Participants shared the experience of losing their normal hearing ability and referred to not being able to hear “*like before*”, albeit to differing degrees. The degree of hearing loss from aminoglycosides varies in the literature; in Harris et al.'s (68) study, 27% of adults with aminoglycoside-related hearing loss presented with mild or moderate hearing loss, none had moderately severe hearing loss and 60% presented with severe or profound hearing loss. In another South African study, approximately 61% of MDR-TB patients had mild or moderate hearing loss, 13% had a moderately-severe hearing loss and 26% had a severe or profound loss (69).

Participants began to realise that their hearing had deteriorated upon finding it increasingly difficult to understand others' speech. For most persons with hearing loss, the primary impact of altered hearing is on speech perception (70). Altered hearing also affects the relationship between what the listener hears and what the listener experiences (70). The two participants with profound hearing loss experienced a disconnect between visual and auditory information:

“*When people talked, I would shout ‘hey, why are you moving your mouth and then you don’t raise your voice up?’*” (Elizabeth)

Upon realising they had hearing loss, participants felt confusion and disbelief in asking themselves “*is this really happening to me?*” and experiencing “*shock that I’m actually losing my hearing*”. Participants also expressed being scared and depressed. Research on hearing loss experienced in adolescence has shown that both depression and anxiety, or both, are present in American hearing impaired AYA, and that the extent of hearing loss (severe to profound) and older age of the participants was associated with higher scores on scales exploring depression and anxiety (71). Findings from a systematic review upheld the notion that hearing impaired children and AYAs had a higher point and lifetime prevalence for anxiety and depression, when compared with community based samples (72).

Despite the high income country setting for the cited research regarding hearing loss in adolescence, these studies, along with signals that quality of life is poorer in AYA with hearing loss compared with normal hearing (73), it would be reasonable to expect that in a challenging LMIC, mental health would be compounded still further. However, is too simplistic to attribute all mental health problems experienced by hearing impaired AYA to hearing loss alone. Having TB in itself raises the odds of depression almost 4-fold, and interestingly, being depressed raised the risk of developing TB in one small study (74). While much chronic illness can precipitate depression, likely the stigma and hardship associated with MDR-TB exacerbates depression still more, with a recent meta-analysis reporting a pooled prevalence of 52.3% (75). Psychological concerns including hopelessness, a sense of worthlessness and suicidal ideation have all been described in MDR-TB patients (65), suggesting chronicity equates to elevated risk. Patwal and colleagues examined rates of suicidal ideation and attempts in a meta-analysis (76). Suicide and attempted suicide were higher in a TB population than a general population, with greater likelihood in females, those being treated for TB repeatedly (MDR), and those with HIV co-infection (76). Thus, health care practitioners should be mindful of psychological challenges in all MDR patients, but particularly in those with an additional burden of hearing loss.

For participants, living with loss of effortless communication was a distressing consequence of hearing loss. Participants had to adapt the way they communicated with the hearing world to prevent or rectify communication breakdown. Adaptations included requests for repetition, informing unfamiliar communication partners of hearing difficulties and/or requirements for more effective communication, using visual cues and using text such as subtitles and text messaging. Participants had to put conscious effort into communicative exchanges; Teekay described having to “*pay attention*” and “*think fast*” when conversing. When individuals experience communication difficulties due to hearing loss, the expectation is that they are the source of the problem and are responsible for developing strategies to conform to the hearing world, in which spoken language is the standard and good hearing is taken for granted. A statement by Hallam et al. (15) that the adaptations necessary to communicate successfully were “bewildering unfamiliar to the newly deafened person” (p. 6) rang true for participants.

All but one participant had received hearing aids to aid communication. Although participants made positive comments about hearing aids, not one made use of a hearing aid. They reported barriers to hearing aid use which included not having a supply of batteries, being embarrassed to wear it, and being afraid that it might be stolen. Negative experiences of hearing aid use include perceived social stigma and increased noise and physical discomfort (77). In a South African study only 12% of adults reported using their hearing aids daily (78). Factors that affected participants' hearing aid use in the current study, such as cost of batteries and cosmetic concerns, resonated with their findings. A participant in a Nigerian study described mourning the loss of his hearing, but hearing needs were unmet, and stopped at a consultation with an ENT (65). Other plausible explanations for limited hearing aid use and benefit include language barriers between audiologists and patients, financial issues, transportation (78), technical problems, monaurally fitted hearing aids and unrealistic expectations (79). While these factors were not specifically reported by participants in this study, they show the many challenges to the use of hearing aids.

While most participants in this study had received hearing aids, the LMIC context should be considered. Waterworth and colleagues lamented the rising levels of hearing loss, lack of hearing health care professionals, and equipment and resources issues concerned with hearing management (80). Aural rehabilitation must go beyond simply supplying a hearing aid, and this has been shown to be challenging in South Africa, where resources including appropriate rehabilitation programme are lacking (81).

Participants were living with fear of permanent and complete hearing loss. A recurring concern was that participants, especially those with lesser degrees of hearing loss, would lose their hearing completely:

“*I thought [I] was going to be completely deaf.*” (Bianca)

Participants questioned whether their hearing would improve; Bianca “*started to worry… would [she] be able to hear one day?*” Others turned to healthcare professionals asking, “*when will we hear?*”. The two participants with profound loss came to the realisation that their hearing loss was permanent:

“*I see that this problem, it can't get off, it’s all forever.*” (Teekay)

While there is no way of predicting the degree to which one's hearing loss will deteriorate, participants' lack of awareness about the permanency of their loss may again be attributed to insufficient counselling about ototoxicity. Khoza-Shangase et al. (40) noted in their study that 63% of the adults who experienced auditory symptoms from TB treatment were told by healthcare workers that their symptoms were a side-effect of the treatment and would diminish after treatment.

Participants had to contend with living with the stigma of hearing loss. Participants not only had to struggle with the stigma of being different due to their TB status, but also because of their hearing status. The experience of discrimination increased their fear of rejection. They believed that they were being perceived differently, or feared that they would be, because of the use of hearing aids:

“*I always felt embarrassed when I wore [the hearing aid] …People always looked at me when I was wearing that thing.*” (Bianca)

Hearing aids replace the disability of hearing loss with that of stigma; hearing aids are fitted to restore normality but in doing so make the user's hearing loss apparent, which might have otherwise gone unnoticed, essentially drawing the attention of others to the user's abilities and inabilities (77). As adolescents are especially concerned with their appearance (82), it is reasonable to speculate that perceived stigma associated with hearing aid use may be amplified in the adolescent years.

Sandisile even refused to disclose her hearing loss for fear of being called a “*disabled person*”. The invisible nature of hearing loss means that affected persons have to decide whether to conceal or disclose their condition (83). Adults' choices to conceal their condition may be attributed to stigma (84) and the expectation of negative judgment (77). Deciding how and when to disclose hearing loss in each interaction is also a complicated process (84).

Participants reflected on living with others’ reaction to hearing loss. The way in which communication partners responded to hearing loss and communication breakdown directly affected participants' experiences. The degree of disability that individuals with hearing loss experience is often affected by others' reactions and attitudes to them; disabling effects are minimised when individuals show awareness and put effort into their interactions and maximised when they are not willing to do so (84). Hallam et al. (84) explored the views and perceptions of general public attitudes of 337 adults with acquired profound hearing loss using questionnaires, focus groups and in-depth interviews. The experiences of adults in their study resonated with participants who also perceived a variety of attitudes towards them (84). Participants reported that some communication partners were not discouraged by communication difficulties and were willing to use written communication:

“*She will come with a pen and paper, and we will write or sometimes we write on her phone if we don’t have a pen.*” (Elizabeth)

Participants were however the source of frustration in other communication partners:

“*My mom and my sister get frustrated with me if I can’t hear them. They tell me something and they have to repeat every time.*” (Liezel)

As a result of the increased effort required by communication partners, Teekay described how some would become impatient and “*…just say, ‘Oh, it’s okay, never mind’*”*.* This was also reported by Hallam et al.'s (84); communication partners became irritated, impatient and annoyed when communication breakdown occurred or when asked to speak louder or repeat themselves (84). Participants in their study also felt that hearing individuals were unwilling to change their communication habits and deliberately excluded them by avoiding them or ending the interaction (84).

Participants were accused of “*cheating*” or “*joking*” about their hearing loss and were ridiculed, and for one participant, even by nurses. Such experiences may be attributed to lack of empathy from communication partners. Adolescents with early-onset hearing loss and adults with acquired hearing loss reported that hearing individuals have poor awareness of the impact of hearing loss on one's life (85) and underestimate its significance (86). They also felt that hearing individuals lack sympathy towards them, which one author attributed to others having minimal experience with individuals with hearing loss (84). Adults with acquired profound hearing loss were dismayed that doctors did not recognise the seriousness of their hearing loss, and felt belittled when they were made to feel that they were a nuisance (87).

Arising from the previous themes, an overarching theme living with the consequences of hearing loss: employment and social challenges emerged. Participants described how hearing loss contributed to limited employment prospects. Before finding employment in a retail store, Liezel “*had concerns about finding work because I just thought, how would I cope with having this hearing problem and working; and would I be able to hear instructions or when someone calls me…?*” Her concerns were not unfounded, as Tye-Murray et al. (88) study reported that adults with hearing loss found that their hearing loss had impaired their job performance, which was attributed to their compromised ability to communicate with others (88). In our study participants were disheartened at being unable to follow career paths they had previously envisioned for themselves:

“*I wanted to be a doctor… if [she] finished school and wasn’t deaf*” (Bianca)

Participants expressed significant challenges socially, and became less socially involved in the hearing world:

“*Before I had the hearing problem, I was active in church. I was singing and partaking in activities.*” (Liezel)

Adults with acquired hearing loss have reported experiencing deterioration in the quantity and quality of social exchanges (86). Such findings are similar among adolescents with early-onset severe to profound hearing loss who achieved lower Social Acceptance and Close Friendship scores compared to hearing peers (89). Another study reported that adolescents with hearing loss had fewer friends than their hearing peers (90).

The two participants with profound hearing loss withdrew from social interactions and turned to more solitary activities such as sleeping, sitting alone, watching television, or reading. They were unable to mutually enjoy activities of the hearing world:

“*You can't hear, so the entertainment is going to be boring if you go with the other guys.*” (Teekay)

Adults with acquired profound hearing have reported physical and emotional isolation (91). Being unable to participate in the hearing world resonated with adults with acquired hearing loss in Barlow et al.'s study (91) who felt that they did not belong in the hearing world nor in the pre-lingually deaf world. They felt that their hearing loss “had left them between worlds, in a twilight zone, and had robbed them of their identity” (p. 445) (91). Adolescents with early onset hearing felt the same, as though they were “trapped between two worlds” (p. 140) (85).

In experiencing significant life change, participants no longer identified with their peer group. Hearing loss differentiated participants from their peers:

“*I feels like my life is different to other people*” (Elizabeth)

They also had to come to terms with their lives being different to how they had envisioned it would be before acquiring hearing loss:

“*I dream that my life would’ve been normal…. If I wasn’t half deaf, then my life wouldn’t have been like this.*” (Bianca)

The experience of loss of participants’ former selves, and the loss of the lives and futures they had was also reported in a study with adolescents who had sustained paraplegia or quadriplegia; they referred to not being able to be the person they were before the injury and feeling ‘different’ (91). The current findings echoed the theme of ‘old self no more, forever changed’ in a study which investigated the experiences of traumatically injured adolescents (93).

## Implications and conclusions

This study described the lived experiences of South African adolescents with acquired hearing loss following aminoglycoside treatment for MDR-TB. It is evident from the findings that social objectives, identity construction, the desire to conform, and independence associated with adolescent life (82) were threatened by the triple burden of living with poor socio-economic circumstances, MDR-TB and hearing loss. The findings contribute to the limited body of research on acquired hearing loss during adolescence and have implications for how health care professionals manage this significant section of South African society.

The findings have numerous implications for management of adolescents with hearing loss. Most participants experienced unfavourable socio-economic circumstances and, as such, AYA in similar circumstances, or their caregivers, should be made aware of social and disability grants (62). Career counselling should be considered for AYA who live with a significant hearing impairment or chronic illness (94). Given that participants experienced profound emotional and social consequences, holistic management should include psychological counselling and social worker involvement. Peer and patient support groups may also be beneficial as they promote emotional (37) and social support (64) and have been recommended for those experiencing bereavement (95), undergoing MDR-TB treatment and with hearing loss (37). Pre-emptive informational counselling about TB and treatment side-effects should form part of the standard of care of MDR-TB patients to alleviate confusion in response to diagnosis and adverse side-effects, and has been demanded by MDR-TB survivors who sustained hearing loss (96). Counselling should include information about the signs and symptoms of ototoxicity, including tinnitus and the permanent nature of ototoxic hearing loss, to alleviate confusion at the onset of auditory disturbances. Counselling of TB patients and their families, and education of communities and schools in areas burdened with TB (36), should be provided in order to improve knowledge and reduce stigma experienced by MDR-TB patients. Individuals who acquire hearing loss, as well as their families, should receive intensive counselling (97) to facilitate adjustment and acceptance of hearing loss (97). Management of communication difficulties must involve timely rehabilitation in the form of hearing aids (68) or cochlear implantation (97). Whether devices are prescribed or not, hearing-impaired patients are entitled to receive a comprehensive and holistic programme of aural rehabilitation. For example, counselling regarding communication strategies should be provided to patients and family members. Lip-reading training for those with profound hearing loss and/or formal speech perception training should also be considered.

Ototoxicity monitoring is a critical part of the package of MDR-TB care (68). While guidelines for assessment and management of ototoxicity were issued by Health Professions Council of South Africa in 2018, adherence to the guidelines by audiologists is suboptimal (98) and utilisation of out-patient ototoxicity monitoring services by patients is poor (99). Well implemented and utilised ototoxic monitoring is crucial as early detection and management of hearing loss may improve quality of life and should be regarded as mandatory. The primary aim of monitoring programs is to prevent noticeable hearing loss by detecting it before it progresses to affect speech frequencies and replacing or discontinuing ototoxic drugs (68). Alteration of treatment regimens safely and effectively may not be possible without compromising cure (68) which highlights the need for safer MDR-TB drugs (100).

By showing the profound and life-changing consequences of aminoglycoside-related hearing loss, the current research serves as motivation for the improvement of current monitoring practices (98) and patient uptake of monitoring services (99) as well as the cessation, or at least cautious use, of aminoglycosides. Since this research was completed, WHO has recommended extensive changes to the regimen for treating MDR-TB, a strategy hailed by survivors of MDR-TB (96), whose harrowing accounts of aminoglycoside-induced hearing loss closely echo the experience of the young participants in this study.

The results of this small qualitative study cannot be generalised but offer an in-depth description into the lived experiences of adolescents with acquired hearing loss and may be transferable to similar populations and settings. The findings were limited to a unique subset of individuals, that is, AYA with acquired hearing loss specifically from MDR-TB treatment living in a developing country. The context of living with limited support and with MDR-TB could not be removed from the experience of acquired hearing loss and thus, the findings may not serve to reflect the impact of acquired hearing loss from other aetiologies or in other contexts.

The recommendations made because of the findings consider the whole person, and not just a person with hearing loss, and promote holistic, patient-centred care. Such recommendations should form part of the standard of care of individuals with acquired aminoglycoside-related hearing loss. The authors close by calling for compassionate and considerate care for MDR-TB patients worldwide.

## Data Availability

The raw data supporting the conclusions of this article will be made available by the authors, without undue reservation.
